# Driving Digital Deprescribing—Enhancing PPI Discontinuation With an App‐Based Strategy

**DOI:** 10.1002/ueg2.70310

**Published:** 2026-10-13

**Authors:** Jacob Hamm, Christoph Ammer‐Herrmenau

**Affiliations:** ^1^ Department of Gastroenterology Gastrointestinal Oncology and Endocrinology University Medical Center Goettingen Goettingen Germany

**Keywords:** deprescribing, healthcare apps, personalized medicine, PPI

## 1 Introduction

Proton pump inhibitors (PPI) are one of the most commonly prescribed drug classes worldwide. However, the problem of overprescription of PPIs is apparent as in up to two thirds of cases, PPIs are prescribed without a justified indication [1]. Chronic PPI intake has been associated with several adverse events such as chronic kidney disease or bone fractures. Most of these associations stem from observational studies and do not prove causal relationships [2]. Nevertheless, if prescribed without being decidedly indicated, PPIs should be discontinued as it should be the case for any other drug.

Several previous publications have assessed various deprescribing strategies for PPIs such as medical information discussions combined with telephone medical consultations [3], patient brochures [4] or even software tools to guide general practitioners to deprescribe PPIs if there use is not indicated [5]. Generally, these interventions have shown the capability to increase the rate of PPI discontinuation to a certain extent. However, an app‐based strategy to even further increase deprescribing efficacy has not yet been tested.

## 2 A Digital and Personalizable Tool to Deprescribe PPIs

The open label adaptive randomized controlled superiority trial performed by Koggel et al. shows that the use of an app‐based intervention can significantly improve PPI deprescribing for the first time. 86% of all patients in the intervention group and 64% of all patients in the control group (brochure information) discontinued PPIs (*p* = 0.002) [6]. In comparison to previous publications, the success rate in the control group was unusually high [4, 5]. This may be attributed to the fact that all invited patients received information on the rationale of the study as well as potential adverse effects of PPIs. The fact that the authors still observed a significant advantage of the app‐based deprescription strategy over the control group in this setting substantiates the efficacy of this tool. Interestingly, the notifications within the app were adjusted to individual preferences were patients could choose between a full stop of PPI use and two different tapering regimes which even allowed for a personalized approach. Symptoms evident of a rebound effect (higher gastric acid secretion after PPI discontinuation) as assessed using the Patient Assessment of Gastrointestinal Symptom Severity Score (PAGI‐SYM) were significantly increased between baseline and 2 month follow up in the intervention group (*p* = 0.023) whereas this was not the case for the control group (*p* = 0.085). However, re‐initiation of PPI intake was slightly lower in the intervention (17%) in comparison to the control group (22%).

## 3 An App for Everyone?

The majority of patients (72%) included in this randomized controlled trial received PPIs for ulcer prophylaxis. In population‐based studies in Europe, ranges between 26.8% and 53.5% among PPI prescriptions are reported to be initiated for ulcer prophylaxis which is a much lower percentage [7, 8]. This raises the question of the extent to which the study's results are applicable to the entire population. Here, a follow‐up study which applies the app to a group of patients which resembles the proportions of PPI indications in the general population more closely would be of significant additional value. Moreover, the reality of an overaged population of patients may hamper the applicability of a smartphone‐based tool as to limited digital competence [9]. As the generation of “digital natives” grows older this may however change.

## 4 A Long Lasting Effect?

The study by Koggel et al. has clearly shown a benefit of app‐based PPI discontinuation during the 2 month follow up. However, how long this effect might be lasting is another question. The data by the present study already suggests that albeit effective in the short term, re‐initiation of PPI intake occurred comparably often in both the intervention (17%) and control group (22%). The median follow‐up was 8 months which is a rather short period of time. Thus, it remains unanswered whether even more patients will re‐initiate PPI therapy later on. Here, a follow‐up study with a longer follow‐up combined with a prolonged period in which patients keep being motivated by the app to abstain from PPI intake would be of substantial merit.

Overall, the presented healthcare app which improves physician–patient interactions by empowering patients to take an active role in implementing medical interventions proves to be effective. Importantly, the present study elegantly demonstrates that the widespread problem of PPI overprescription can be addressed using an innovative digital tool.

## Conflicts of Interest

The authors declare no conflicts of interest.

## Data Availability

The authors have nothing to report.
